# Characteristics of immunoglobulin G4-related aortitis/periaortitis and periarteritis on fluorodeoxyglucose positron emission tomography/computed tomography co-registered with contrast-enhanced computed tomography

**DOI:** 10.1186/s13550-017-0268-1

**Published:** 2017-02-27

**Authors:** Satoshi Yabusaki, Noriko Oyama-Manabe, Osamu Manabe, Kenji Hirata, Fumi Kato, Noriyuki Miyamoto, Yoshihiro Matsuno, Kohsuke Kudo, Nagara Tamaki, Hiroki Shirato

**Affiliations:** 1 Department of Radiation Medicine, Hokkaido University Graduate School of Medicine, N15 W7, Kita-Ku, Sapporo, 060-8638 Japan; 2 Department of Diagnostic and Interventional Radiology, Hokkaido University Hospital, N14 W5, Kita-Ku, Sapporo, 060-8648 Japan; 3 Department of Nuclear Medicine, Hokkaido University Hospital, N14 W5, Kita-Ku, Sapporo, 060-8648 Japan; 4 Department of Surgical Pathology, Hokkaido University Hospital, N14 W5, Kita-Ku, Sapporo, 060-8648 Japan

**Keywords:** FDG-PET/CT, Contrast-enhanced CT, IgG4-related disease, IgG4-related vascular disease

## Abstract

**Background:**

We aimed to assess the positivity, distribution, quantitative degree of vessel inflammation, and clinical characteristics of IgG4-related aortitis/periarteritis and periarteritis (IgG4-aortitis), and to examine the difference in these characteristics between cases with and without IgG4-aortitis, using fluorodeoxyglucose positron-emission tomography/computed tomography (FDG-PET/CT) co-registered with contrast-enhanced CT (CECT).

We retrospectively evaluated 37 patients with IgG4-related disease (IgG4-RD) who underwent both FDG-PET/CT and CECT. The arterial SUVmax and its value normalized to the background venous blood pool (BP)—the target-to-background ratio (TBR) in the entire aorta and the major first branches—were measured. Active vascular inflammation was considered in cases with a higher FDG uptake than BP and a thickened arterial wall (>2 mm).

**Results:**

Fifteen (41%) patients exhibited IgG4-aortitis. Most patients (80%) showed multiple region involvement. The entire aorta, including the major first branches, were involved, typically showing a thickened wall and high FDG uptakes. The most common site was the iliac arteries (35%), followed by the infrarenal abdominal aorta (33%), thoracic aorta (8%), first branches of the thoracic aorta (8%), suprarenal abdominal aorta (6%), and the first branches of the abdominal aorta (5%). The IgG4-aortitis-positive vessel regions were thickened, with an average maximal wall thickness of 6.3 ± 2.9 mm. The SUVmax and TBR values were significantly higher in the IgG4-aortitis-positive regions (median 3.7 [1.6–5.5] and 2.1 [1.4–3.7], respectively) than in the IgG4-aortitis-negative regions (median 2.1 [1.2–3.7] and 1.3 [0.9–2.3], respectively; *p* < 0.0001). The IgG4-aortitis-positive group patients were older (69.5 ± 6.0 vs. 63.3 ± 12.6 years, respectively) and had a higher male predominance (80 vs. 55%, respectively) than the negative group, although the differences were not significant (*p* = 0.17 and *p* = 0.06, respectively).

**Conclusions:**

We investigated the image characteristics of IgG4-aortitis. The entire aorta and major branches can be involved with more than 2-fold higher FDG uptake than the venous background pool, and with wall thickening. The most common involved site is the iliac arteries, followed by the infrarenal abdominal aorta.

## Background

Immunoglobulin G4-related disease (IgG4-RD) is a newly recognized disease entity characterized by the infiltration of IgG4-positive plasma cells and fibrosis in systemic organs along with the elevation of serum IgG4 levels [1]. Autoimmune pancreatitis and Mikulicz’s disease are representative phenotypes, and lymph node swelling is a common finding in such cases. Moreover, the pituitary gland, dura mater, orbital cavities, paranasal sinuses, thyroid gland, breast, lung, liver, biliary system, kidneys, prostate, retroperitoneum, and skin are potential targets for disease involvement [2–7].

Vascular involvement has also been detected in IgG4-RD [8, 9]. The features of IgG4-related aortitis/periaortitis and periarteritis (IgG4-aortitis) on computed tomography (CT) include arterial wall thickening and homogeneous wall enhancement [4, 9]. The dissection of the great vessels and myocardial ischemia due to coronary artery involvement are fatal manifestations of IgG4-RD [10–19]. Nevertheless, the prevalence, detailed distribution, and degree of inflammation in IgG4-aortitis have not been reported.

In patients with IgG4-RD, whole-body organ involvement manifests as increased FDG uptake in multiple organs on fluorodeoxyglucose (FDG) positron-emission tomography (PET) and FDG-PET/computed tomography (CT) [20–26]. Although histopathological examination remains the gold standard for detecting organ involvement and diagnosing IgG4-RD, it is difficult to obtain biopsy or surgical specimens from the arterial wall. Hence, non-invasive evaluation of vascular involvement is clinically important not only for diagnosis but also for the management of IgG4-aortitis. FDG-PET/CT has also been reportedly used to assess other inflammatory vascular diseases, such as Takayasu arteritis and giant cell arteritis [27, 28]. However, to our knowledge, no study has described the use of systemic evaluation using FDG-PET/CT co-registered with contrast-enhanced CT (CECT) for IgG4-aortitis.

In the present study, we aimed to assess the positivity, distribution, quantitative degree of vessel inflammation, and clinical characteristics of IgG4-aortitis, and to examine the differences in these characteristics between cases with and without IgG4-aortitis on FDG-PET/CT co-registered with CECT.

## Methods

### Patients

This retrospective study was approved by the institutional review board. The requirement for obtaining informed consent was waived. Forty-three patients who were diagnosed with definite IgG4-RD according to the 2011 comprehensive diagnostic criteria for IgG4-RD proposed by Umehara et al. [3] and who underwent FDG-PET/CT and CECT within 3 months of diagnosis, from January 2009 to July 2015, were screened. Five patients who had received steroid therapy prior to FDG-PET/CT and CECT were excluded, and one patient was excluded because of high serum glucose levels, exceeding 200 mg/dL. Hence, 37 patients were finally included in the study. It was confirmed that none of the patients had untreated or recurrent malignant disease during the follow-up period (median 56 months, range 9–92 months).

The background of the patients is summarized in Table 1. The median patient age was 68 years (range 39–83 years), and 24 patients were male. The median interval between the applications of the two modalities was 13 days (range 1–88 days). All patients underwent biopsy or surgery, and 22 patients exhibited IgG4-positive plasma cell infiltration. There were 15 patients with negative histopathological diagnoses. However, eight of these patients were diagnosed according to the Clinical Diagnostic Criteria for Autoimmune Pancreatitis of Japan 2006 [29], and the other seven patients were diagnosed according to the Diagnostic Criteria for IgG4-positive Mikulicz’s disease [30]. All patients showed abnormal increases in serum IgG4 levels, exceeding 135 mg/dL, except for one (patient #17) with normal IgG4 levels diagnosed as having definite IgG4-RD according to the Clinical Diagnostic Criteria for Autoimmune Pancreatitis of Japan 2006 [29].

**Table 1:** Patient characteristics

No.	Age (y)	Sex	Diagnostic criteria	Positive histopathological site	Interval between CECT and FDG-PET/CT(d)	Serum IgG4 value (mg/dL)	Vascular involvement	Other positive site
1	79	F	Definite	Pancreas	38	N/A	+	LN, SG, P
2	75	M	Definite	Minor salivary gland	7	953	+	LN
3	61	M	Definite	Minor salivary gland, bile duct	7	968	+	LN
4	70	M	Definite	Gall bladder	9	802	+	LN, P
5	67	M	Definite	Nasal mucosa	1	1390	+	LN, SG, C
6	73	M	Definite	Bile duct	17	1370	+	LN, SG, C, P
7	74	M	Definite	Lung	21	384	+	LN, SG, C
8	61	F	Definite	Pancreas	12	149	+	LN, SG, P
9	71	M	Definite	−	43	N/A	+	LN, P
10	77	M	Definite	−	6	N/A	+	LN
11	69	M	Definite	−	6	733	+	LN, SG
12	71	M	Definite	−	8	469	+	LN, SG, P
13	68	F	Definite	−	13	571	+	LN, P
14	68	M	Definite	−	28	877	+	LN, SG, C, P
15	58	M	Definite	−	12	326	+	LN, SG, P
16	78	M	Definite	Abdominal aorta	3	225	−	P
17	77	M	Definite	Bile duct	8	89.8	−	LN, P
18	54	M	Definite	Nasal mucosa, bile duct	6	455	−	LN, SG, P
19	73	F	Definite	Lacrimal gland	28	N/A	−	LN
20	55	F	Definite	Minor salivary gland	80	N/A	−	SG
21	60	F	Definite	Nasal mucosa	26	N/A	−	LN, SG
22	78	M	Definite	Minor salivary gland, nasal mucosa	11	888	−	LN, SG
23	50	M	Definite	Nasal mucosa	5	408	−	LN, P
24	67	M	Definite	Nasal mucosa	13	377	−	^a^
25	51	F	Definite	Lacrimal gland	1	398	−	LN, SG
26	45	M	Definite	Submandibular gland	20	N/A	−	LN, C
27	83	M	Definite	Minor salivary gland	49	905	−	LN, SG
28	78	F	Definite	Pancreas	31	243	−	LN, P
29	39	F	Definite	Lymph node	3	479	−	LN, SG
30	75	M	Definite	−	88	N/A	−	^b^
31	73	F	Definite	−	56	N/A	−	LN, SG
32	66	M	Definite	−	32	N/A	−	C
33	59	F	Definite	−	8	N/A	−	LN, SG, C, P
34	67	M	Definite	−	33	526	−	C, P
35	60	M	Definite	−	23	862	−	LN, SG
36	46	F	Definite	−	30	797	−	LN, SG
37	58	F	Definite	−	60	977	−	LN, SG

*Y* year, *d* days, *M* male, *F* female, + positive, − negative, *N/A* not available, *LN* lymph node, *SG* salivary gland, *C* chest (pleura/paravertebra), *P* pancreas

^a^(Patient no. 24) A patient with small hypovascular lesions in the bilateral kidney on CECT; the FDG-PET/CT findings could not be assessed due to physiological uptake by the renal parenchyma. This patient was treated for IgG4-RD

^b^(Patient no. 30) A patient with abnormal pancreas findings on CECT, without significant abnormal FDG uptake. The patient was diagnosed as having autoimmune pancreatitis using endoscopic retrograde cholangiopancreatography

The serum IgG4 levels within 2 months from PET/CT were available in 28 patients.

### Imaging techniques

FDG-PET/CT was performed using a hybrid scanner (Biograph 64 with TrueV and high-definition PET, Siemens Japan, Tokyo, Japan), with lutetium oxyorthosilicate crystal detectors, covering an axial field of view of 21.6 cm, and a 64-slice multi-detector computed tomography scanner. The patients were instructed to fast for more than 6 h, and their blood glucose level was measured (median blood glucose level, 101 mg/dL; range 79–182 mg/dL) before injecting the FDG. A mean total dose of 240 ± 35 MBq of the FDG was administered intravenously, which was computed as 4.2 ± 0.6 MBq/kg. Following the injection, the patient was allowed to rest comfortably for 60 min. Image acquisition was initiated with a non-contrast-enhanced CT scan for attenuation correction and co-registration. This was followed by three-dimensional PET acquisition, with a 3-min time-limit per position, from the top of the skull to the mid-thigh.

Standard diagnostic CT scans were performed with a 64-multislice CT scanner (Aquilion 64, Aquilion PRIME, or Aquilion One ViSION edition, Toshiba Medical Systems, Otawara, Japan; LightSpeed VCT, GE Healthcare, Milwaukee, WI, USA; or SOMATOM Sensation 64, Siemens AG Medical Solutions, Erlangen, Germany). The CECT protocol in the present study included a single-phase scan performed from the neck to the pelvis, 70–180 s after the injection of contrast material (450–560 mg·I/kg), with a 5-mm section thickness.

### Imaging analysis

Vascular involvement was assessed at the following six regions: thoracic aorta, suprarenal abdominal aorta, infrarenal abdominal aorta, iliac arteries (common, external, and internal iliac arteries), first branches of the thoracic aorta (brachiocephalic, left common carotid, and left subclavian arteries), first branches of the abdominal aorta (celiac, superior mesenteric, renal, and inferior mesenteric arteries).

**Fig. 1:**
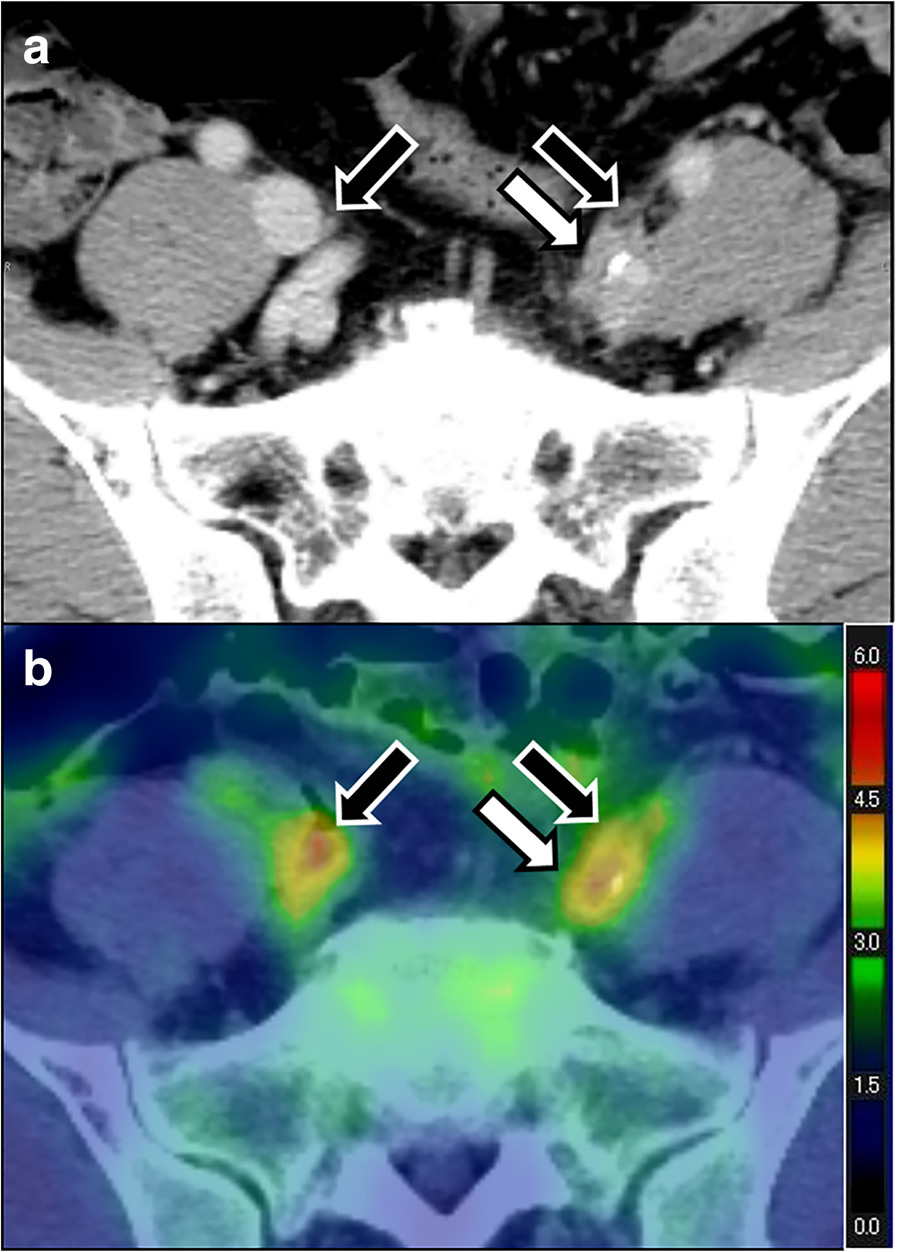
CECT (**a**) and FDG-PET/CT (**b**) images demonstrating left internal iliac artery involvement (*white arrow*). Physiological uptake by the urinary tracts (*black arrows*) cannot be differentiated using FDG-PET/CT alone

Of the 222 vascular regions in 37 patients, 220 regions were evaluated; two regions could not be assessed due to suprarenal and infrarenal aortic replacement. Below the kidney level, abnormal vessel FDG uptake was differentiated from uptake in the urinary tract using co-registered CECT images (Fig. 1).

FDG-PET/CT and CECT images were simultaneously reviewed by two board-certified radiologists (one with 10 years of radiology experience and another with 20 years of experience and a certification in nuclear medicine) in consensus.

The diagnostic criteria for CECT were based on the findings of a previous study [4, 9, 31, 32]. For active inflammatory arteritis, abnormal diffuse arterial wall thickening (>2.0 mm) [31] and homogeneous enhancement on CECT [4, 9, 32] were considered to indicate positive involvement. Since IgG4-aortitis revealed severe inflammatory cell infiltration, mainly into the adventitia [9], the maximal radial vessel wall thickness measurement was carefully performed in such a way as to avoid including intimal atherosclerotic calcification. The presence of aneurysmal changes or dissection was recorded, and maximum wall thickness was measured on CECT axial images.

The diagnostic criteria of the FDG-PET/CT were based on visual evaluation of a higher abnormal vessel wall uptake than that in the blood pool. For qualitative evaluation, the vessel standardized uptake value (SUVmax) of each vascular region was defined as the maximal value by drawing several volumes of interest (VOIs) with a radius of 5 mm. For normalizing the SUVmax value, we also calculated the ratio of the arterial SUVmax to that of the venous blood pool, also known as the target-to-background ratio (TBR). Rudd et al. described the TBR as a reproductive and quantitative method for assessing the arterial wall [33]. Besson et al. reported the utility of TBR for the assessment of aortic inflammation in giant cell arteritis patients [34]. With regard to the background, the VOIs in the inferior vena cava and right atrium were also measured. The SUVmax of the background venous blood pool was calculated as the mean value of the SUVmax at the right atrium and inferior vena cava.

When abnormal findings were simultaneously detected on both CECT and FDG-PET/CT images, the region was considered to have positive inflammation due to IgG4-aortitis (IgG4-aortitis-positive region). IgG4-aortitis was differentiated from atherosclerosis as follows: if there was an abnormal finding only with CECT or FDG-PET/CT, or if there was no abnormal finding on both CECT and FDG-PET/CT, the region was considered as IgG4-aortitis-negative region. This sorting was applied to six regions independently in each patient. If a patient had at least one positive vascular region, the patient was assigned to the IgG4-aortitis-positive group. Patients without vascular involvement were assigned to the IgG4-aortitis-negative group. The involvement of other organs, such as lymph node, salivary gland, pancreas, and chest (pleura and paravertebral region) were simultaneously assessed. The diagnostic clue for lymph node involvement is abnormal swelling and diffuse enhancement. The salivary gland shows unilateral or bilateral diffuse or focal swelling and homogeneous enhancement. The pancreas shows diffuse or focal enlargement with irregular narrowing and loss of the pancreatic cleft and smooth rim (capsule-like rim). Pleural and paravertebral involvements are indicated by thickened or flattened soft tissue density areas along the pleura and vertebra [4, 5, 32, 35]. The lacrimal gland and paranasal sinuses were not assessed, since the scanning range of whole-body CECT did not include it in all cases. The kidneys could not be assessed due to the physiological uptake in the renal parenchyma.

### Statistical analysis

To evaluate the disease distribution, the positivity ratio was calculated for each of the six regions.

We compared the differences in the patient’s background (including sex, age, prevalence of diabetes mellitus, and serum IgG4 level) between the IgG4-aortitis-positive and IgG4-aortitis-negative groups. The categorical variables, including male-to-female ratio and diabetes mellitus, were compared using Fisher’s exact test. An unpaired Student *t* test was used to compare the continuous variables with normal distribution, including age and serum IgG4 levels, between the two groups.

For region-based comparisons, the vessel SUVmax and TBR values were compared between the IgG4-aortitis-positive and IgG4-aortitis-negative regions. Since the vessel SUVmax and TBR values were not normally distributed, they were compared using the nonparametric Wilcoxon rank-sum test.

Continuous variables are presented as mean ± standard deviation or as median (range), whereas categorical variables are presented as percentages. A *p* value <0.05 was considered significantly different.

All statistical analyses were performed using dedicated software JMP 12.2.0 (SAS institute, Cary, NC, USA).

## Results

### Positivity, distribution, and clinical characteristics of IgG4-aortitis

Fifteen of 37 patients (41%) had at least one positive IgG4-aortitis-positive region and were assigned to the IgG4-aoritis-positive group (Table 1); among these patients, three (20%) exhibited vascular involvement at a single site and 12 (80%) exhibited vascular involvement at multiple sites. A total of 35 IgG4-aortitis-positive regions were detected, and the average number of positive vessels per patient was 2.3. The detailed distribution, maximal wall thickness, and vessel TBR of the IgG4-aortitis-positive regions in these 15 patients are summarized in Table 2.

**Table 2:** Summary of vascular lesions

No.	TBR						Maximal wall thickness (mm)	Total number of positive vascular regions
	TA	Suprarenal AA	Infrarenal AA	IA	TA-branch	AA-branch		
1	−	−	−	2.7	−	3.6^a^	7.9	2
2	−	−	3.7	2.8^a^	−	−	9.8	2
3	−	−	1.6^a^	1.6	−	−	3.0	2
4	−	−	2.6^a^	2.2	1.8	−	4.7	3
5	−	−	2.0^a^	1.7	−	−	3.1	2
6	−	−	1.4	2.0^a^	−	−	6.1	2
7	−	−	2.1^a^	2.3	−	−	4.4	2
8	−	−	−	2.6^a^	−	−	3.9	1
9	−	2.2	2.7^a^	1.8	3.0	−	11.3	4
10	1.5^a^	−	2.1	2.1	−	−	4.3	3
11	2.0	2.4	2.4	2.1	1.4^a^	−	5.8	5
12	−	−	1.5	1.7^a^	−	−	8.1	2
13	−	−	−	−	−	1.7^a^	2.5	1
14	1.7	−	2.6^a^	2.2	−	−	11.1	3
15	−	−	2.3^a^	−	−	−	8.1	1
Average	1.7	2.3	2.3	2.1	2.1	2.7	6.3	2.3

*TBR* target-to-background ratio, *AA* abdominal aorta, *IA* iliac arteries, *TA* thoracic aorta, − negative

^a^Region of the maximal wall thickness

**Fig. 2:**
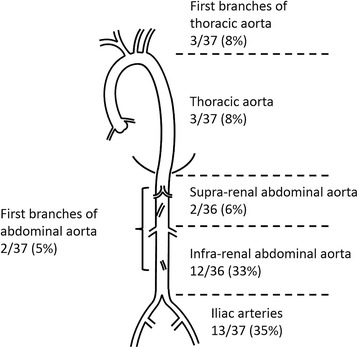
Whole body distribution and positivity of vascular involvement. Fifteen patients exhibited vascular involvements in 35 regions

Figure 2 shows the positivity in each of the six vascular regions. Thirteen of 37 patients (35%) exhibited iliac artery involvement, which was the most common site, followed by the infrarenal abdominal aorta (33%), thoracic aorta (8%), first branches of the thoracic aorta (8%), suprarenal abdominal aorta (6%), and first branches of the abdominal aorta (5%).

**Fig. 3:**
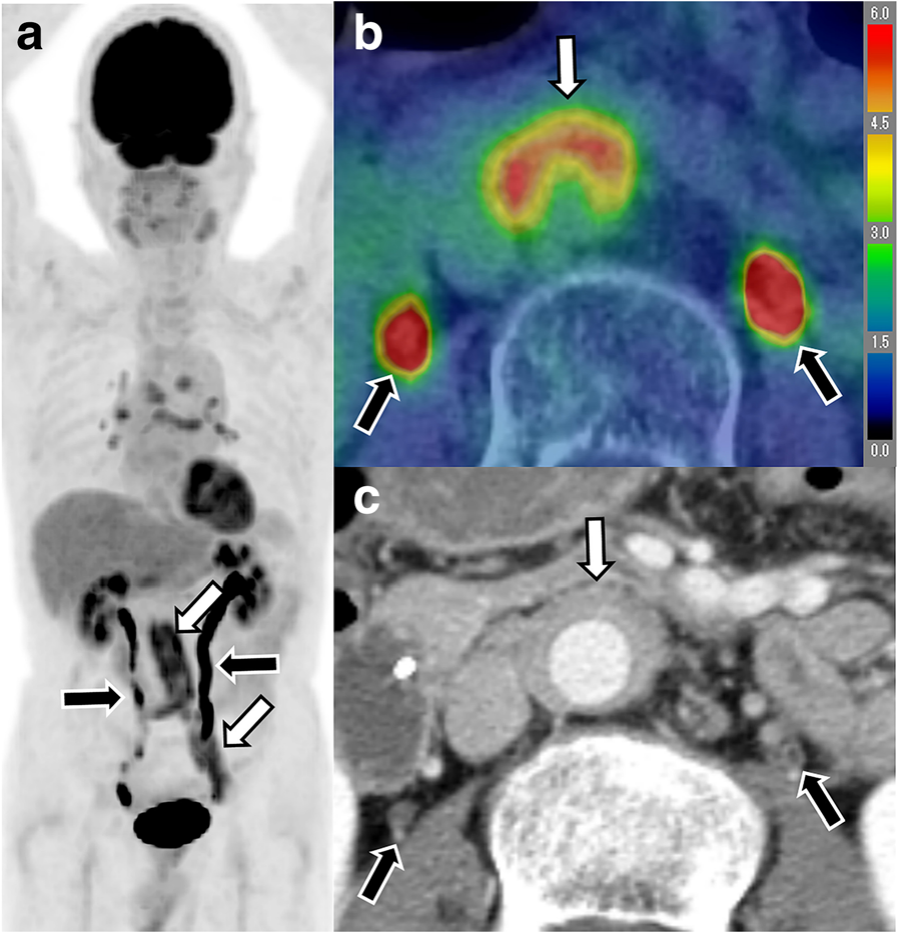
A 75-year-old man with involvement of the abdominal aorta (case #2). MIP image (**a**) showing multiple organ involvement, including hilar lymph node, abdominal aorta, and the left iliac artery (*white arrows*). FDG PET/CT fusion image (**b**) and CECT (**c**) exhibiting marked wall thickening of the abdominal aorta and abnormal FDG uptake (*white arrow*). Note the physiological uptakes in the bilateral urinary tract (*black arrows*)

The IgG4-aortitis-positive vessel regions were thickened, with an average maximal wall thickness of 6.3 ± 2.9 mm. Ten (27%) of 37 patients showed aneurysmal aortic dilatations. Nine of 37 patients (24%) exhibited aneurysmal changes in the iliac arteries, and four patients (11%) exhibited abdominal aortic aneurysms, while three patients showed overlaps between these regional involvements. There was no case of aortic dissection. A representative case with abdominal aorta involvement is described in Fig. 3.

The IgG4-aortitis-positive group was older (69.5 ± 6.0 vs. 63.3 ± 12.6 years, respectively) and had a higher male predominance (80 vs. 55%, respectively) than those in the negative group, although the differences were not significant (*p* = 0.17 and *p* = 0.06, respectively). There was no significant difference in the incidence of diabetes mellitus between the groups (40 vs. 23%; *p* = 0.22). The mean serum IgG4 levels (749 ± 392 mg/dL) in the IgG4-aortitis-positive group were higher than those in the IgG4-aortitis-negative group (545 ± 288 mg/dL), although the difference was not significant (*p* = 0.15).

### Quantification of inflammation in IgG4-aortitis

**Fig. 4:**
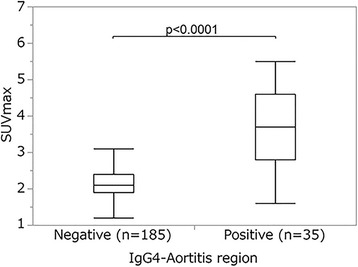
Comparisons of the highest SUVmax values between regions with and without vascular involvement

The median vessel SUVmax value in the IgG4-aortitis-positive regions (3.7; range, 1.6–5.5) was significantly higher than that in the IgG4-aortitis-negative regions (2.1; range, 1.2–3.7; *p* < 0.0001; Fig. 4).

**Fig. 5:**
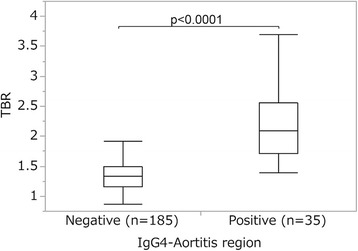
Comparisons of TBR values between patient regions with and without vascular involvement

The median TBR value in the IgG4-aortitis-positive regions (median, 2.1; range, 1.4–3.7) was significantly higher than that in the IgG4-aortitis-negative regions (median, 1.3; range, 0.9–2.3; *p* < 0.0001; Fig. 5).

## Discussion

In the present study, we assessed the positivity, distribution, quantitative degree of vessel inflammation, and clinical characteristics of IgG4-aortitis, and examined the differences in these characteristics between cases with and without IgG4-aortitis using FDG-PET/CT co-registered with CECT. To our knowledge, a detailed systemic assessment of IgG4-aortitis with FDG-PET/CT and CECT has not been reported to date. Approximately, 40% of IgG4-RD patients exhibited vascular involvement, with a high SUVmax and TBR value (median, 3.7 and 2.1, respectively) and wall thickening (median, 6.3 mm). IgG4-aortitis-positive regions exhibited more than 2-fold the FDG uptake of the background blood pool (median, 3.7 vs. 1.6). The typical finding of IgG4-aortitis-positive group was multiple vascular region involvements (80%). The entire aorta and major branches can be involved with more than 2-fold the FDG uptake of the venous background pool. The most common site was the iliac arteries, followed by the infrarenal abdominal aorta.

To assess IgG4-RD, modalities such as CT, magnetic resonance imaging, ultrasonography, Gallium-67 scintigraphy, endoscopic retrograde cholangiopancreatography, endoscopic ultrasonography, intraductal ultrasonography, and FDG-PET or FDG-PET/CT have been used individually or in combination for both morphological and functional evaluation [2, 32, 36–39]. Some authors have reported the clinical applicability of FDG-PET/CT as a single modality in IgG4-related autoimmune pancreatitis [23, 40]. However, FDG uptake is non-specific for vascular evaluation and does not always allow differentiation between atherosclerotic changes and active inflammation [41, 42]. As vascular involvement is more commonly observed in older male patients, it is clinically important to differentiate between IgG4-aortitis and atherosclerosis. Although the evaluation of vessel wall thickness on CECT may serve as a good predictor of vascular inflammation [9, 31], increased wall thickness can persist for a long time after the acute phase of arterial inflammation, thus limiting its relevance in the early assessment of inflammation. Therefore, we investigated the usefulness of a combination of quantitative vessel SUVmax/TBR on FDG-PET/CT and anatomical abnormal wall thickening on CECT. Rudd et al. reported that the mean and maximum TBR values were reproducible and optimal for evaluation of the arterial FDG uptake [33]. Hence, TBR could add to the diagnostic performance, which currently includes visual assessment alone, and may be particularly useful for differentiation from atherosclerosis. Combining the metabolic information from FDG-PET/CT with the thickened vessel wall data from co-registered CECT could be a powerful method for evaluating inflammatory IgG4-aortitis. Moreover, the quantitative vessel SUVmax and TBR values may reduce observer bias as compared to that noted with visual qualitative evaluation of vascular involvement.

Previous studies have reported the prevalence of vascular involvement as 14–23% [24, 25, 32, 43]. However, these studies did not use specific quantitative analysis for active inflammation of the aorta, and the diagnostic criteria for IgG4-aortitis were also not clearly defined. Moreover, these studies did not differentiate between atherosclerosis and IgG4-aortitis. In our study, we quantified abnormal vascular FDG uptake, co-registered with CECT, to differentiate the condition from atherosclerosis or physiological uptake, such as that noted in the urinary tracts. Combination of FDG-PET and CECT may have led to a relatively higher prevalence (41%) of IgG4-aortitis in the present study than in previous reports.

There have been two previous studies that reported the distribution of IgG4-aortitis. Inoue et al. reported that 13 (76%) of 17 patients had infrarenal abdominal aorta or iliac arteries involvement, while the thoracic aorta was involved only in four patients (24%) [9]. This result was concordant with our result. On the other hand, the distribution in a report by Perugino et al. was different [44]. In their 13 patients, eight patients (62%) had thoracic aorta, four patients (31%) with abdominal aorta, and one patient (8%) with iliac artery involvement. The reason for this discrepancy is not clear; however, racial differences may potentially influence the result.

Some form of aneurysmal change was observed in the aortas of 10 patients (27%) in our study, while Perugino et al. reported that 11 of 160 (7%) patients demonstrated thoracic or abdominal aortic aneurysms [44]. These data revealed that IgG4-aortitis carried the potential risk of aortic aneurysm. Although the risk of aortic rupture is unclear, there was no case of dissection in our study. Mizushima et al. reported that a small population of patients with IgG4-aortitis exhibited luminal dilatation after corticosteroid therapy [45]. Hence, patients with IgG4-aortitis should be carefully followed before and after steroid therapy.

In our study, patients with IgG4-aortitis tended to be older and male as compared to those in the negative group, which was also consistent with the previous report [44].

The present study has certain limitations. First, the study design was retrospective in nature, which may have led to possible selection bias and the restriction of evaluable regions. Moreover, we included only newly diagnosed patients, who had not been exposed to immunosuppressive treatments, and hence, not all the patients diagnosed with IgG4-RD were enrolled in this study. Second, the evaluable region may have been restricted due to the non-ECG-gated body CECT scanning protocol; hence, we could not evaluate the head and neck region, and particularly the carotid arteries. The coronary arteries were also not evaluated due to motion artefacts. Third, FDG-PET/CT and CECT images were simultaneously reviewed for division into IgG4-aortitis-positive and IgG4-aortitis-negative regions. Because both a thickened arterial wall on CECT and a higher uptake of FDG in the visual assessment were used for determining IgG4-aortitis-positivity, there was a potential bias that the positive region would show a higher SUVmax value. The IgG4-aortitis-positive region may include both active inflammation and atherosclerosis; however, our aim was not to differentiate active inflammation from atherosclerosis, but to examine the degree of FDG uptake in active regions. Finally, we did not perform biopsies for vascular involvement, as there is no pathological gold standard for diagnosis. Moreover, it is difficult, and may not be safe, to obtain vessel wall specimens. Hence, a non-invasive method is required for the diagnosis of IgG4-aortitis.

## Conclusions

We assessed the positivity and distribution of vascular involvement in IgG4-RD using FDG-PET/CT co-registered with CECT. The entire aorta and major branches may be involved, with more than twice the FDG uptake as compared to the venous blood pool. The infrarenal abdominal aorta and iliac arteries were the most commonly involved sites.

## Abbreviations

IgG4-aortitis: IgG4-related aortitis/periaortitis and periarteritis
IgG4-RD: Immunoglobulin G4-related disease
SUVmax: Standardized uptake value
TBR: Target-to-background ratio
VOIs: Volumes of interest

## References

[1] Hamano H, Kawa S, Horiuchi A, Unno H, Furuya N, Akamatsu T (2001). High serum IgG4 concentrations in patients with sclerosing pancreatitis. N Engl J Med.

[2] Okazaki K, Uchida K, Miyoshi H, Ikeura T, Takaoka M, Nishio A (2011). Recent concepts of autoimmune pancreatitis and IgG4-related disease. Clin Rev Allergy Immunol.

[3] Umehara H, Okazaki K, Masaki Y, Kawano M, Yamamoto M, Saeki T (2012). Comprehensive diagnostic criteria for IgG4-related disease (IgG4-RD), 2011. Mod Rheumatol.

[4] Horger M, Lamprecht HG, Bares R, Spira D, Schmalzing M, Claussen CD (2012). Systemic IgG4-related sclerosing disease: spectrum of imaging findings and differential diagnosis. AJR Am J Roentgenol.

[5] Stone JH, Zen Y, Deshpande V (2012). IgG4-related disease. N Engl J Med.

[6] Umehara H (2012). A new clinical entity: IgG4-related disease (IgG4-RD) discovered in the 21st century. Intern Med.

[7] Hedgire SS, McDermott S, Borczuk D, Elmi A, Saini S, Harisinghani MG (2013). The spectrum of IgG4-related disease in the abdomen and pelvis. AJR Am J Roentgenol.

[8] Tajima M, Nagai R, Hiroi Y (2014). IgG4-related cardiovascular disorders. Int Heart J.

[9] Inoue D, Zen Y, Abo H, Gabata T, Demachi H, Yoshikawa J (2011). Immunoglobulin G4-related periaortitis and periarteritis: CT findings in 17 patients. Radiology.

[10] Kasashima S, Zen Y, Kawashima A, Endo M, Matsumoto Y, Kasashima F (2010). A clinicopathologic study of immunoglobulin G4-related sclerosing disease of the thoracic aorta. J Vasc Surg.

[11] Holmes BJ, Delev NG, Pasternack GR, Halushka MK (2012). Novel cause of sudden cardiac death: IgG4-related disease. Circulation.

[12] Stone JR (2011). Aortitis, periaortitis, and retroperitoneal fibrosis, as manifestations of IgG4-related systemic disease. Curr Opin Rheumatol.

[13] Kasashima S, Zen Y (2011). IgG4-related inflammatory abdominal aortic aneurysm. Curr Opin Rheumatol.

[14] Bito Y, Sasaki Y, Hirai H, Hosono M, Nakahira A, Suehiro Y (2014). A surgical case of expanding bilateral coronary aneurysms regarded as immunoglobulin G4-related disease. Circulation.

[15] Tanigawa J, Daimon M, Murai M, Katsumata T, Tsuji M, Ishizaka N (2012). Immunoglobulin G4-related coronary periarteritis in a patient presenting with myocardial ischemia. Hum Pathol.

[16] Ishizaka N (2014). A suspected case of coronary periarteritis due to IgG4-related disease as a cause of ischemic heart disease. Forensic Sci Med Pathol.

[17] Delgado-Garcia G, Sanchez-Salazar S, Rendon-Ramirez E, Castro-Medina M, Saenz-Ibarra B, Barboza-Quintana A (2016). Myocardial ischemia as presenting manifestation of IgG4-related disease: a case-based review. Clin Rheumatol.

[18] Keraliya AR, Murphy DJ, Aghayev A, Steigner ML (2016). IgG4-related disease with coronary arteritis. Circ Cardiovasc Imaging.

[19] Patel NR, Anzalone ML, Buja LM, Elghetany MT (2014). Sudden cardiac death due to coronary artery involvement by IgG4-related disease: a rare, serious complication of a rare disease. Arch Pathol Lab Med.

[20] Nakajo M, Jinnouchi S, Fukukura Y, Tanabe H, Tateno R (2007). The efficacy of whole-body FDG-PET or PET/CT for autoimmune pancreatitis and associated extrapancreatic autoimmune lesions. Eur J Nucl Med Mol Imaging.

[21] Sato M, Okumura T, Shioyama Y, Imura J (2008). Extrapancreatic F-18 FDG accumulation in autoimmune pancreatitis. Ann Nucl Med.

[22] Matsubayashi H, Furukawa H, Maeda A, Matsunaga K, Kanemoto H, Uesaka K (2009). Usefulness of positron emission tomography in the evaluation of distribution and activity of systemic lesions associated with autoimmune pancreatitis. Pancreatology.

[23] Zhang J, Shao C, Wang J, Cheng C, Zuo C, Sun G (2013). Autoimmune pancreatitis: whole-body 18F-FDG PET/CT findings. Abdom Imaging.

[24] Zhang J, Chen H, Ma Y, Xiao Y, Niu N, Lin W (2014). Characterizing IgG4-related disease with (1)(8)F-FDG PET/CT: a prospective cohort study. Eur J Nucl Med Mol Imaging.

[25] Ebbo M, Grados A, Guedj E, Gobert D, Colavolpe C, Zaidan M (2014). Usefulness of 2-[18F]-fluoro-2-deoxy-D-glucose-positron emission tomography/computed tomography for staging and evaluation of treatment response in IgG4-related disease: a retrospective multicenter study. Arthritis Care Res (Hoboken).

[26] Takahashi H, Yamashita H, Morooka M, Kubota K, Takahashi Y, Kaneko H (2014). The utility of FDG-PET/CT and other imaging techniques in the evaluation of IgG4-related disease. Joint Bone Spine.

[27] Kobayashi Y, Ishii K, Oda K, Nariai T, Tanaka Y, Ishiwata K (2005). Aortic wall inflammation due to Takayasu arteritis imaged with 18F-FDG PET coregistered with enhanced CT. J Nucl Med.

[28] Soussan M, Nicolas P, Schramm C, Katsahian S, Pop G, Fain O (2015). Management of large-vessel vasculitis with FDG-PET: a systematic literature review and meta-analysis. Medicine (Baltimore).

[29] Okazaki K, Kawa S, Kamisawa T, Naruse S, Tanaka S, Nishimori I (2006). Clinical diagnostic criteria of autoimmune pancreatitis: revised proposal. J Gastroenterol.

[30] Masaki Y, Sugai S, Umehara H (2010). IgG4-related diseases including Mikulicz’s disease and sclerosing pancreatitis: diagnostic insights. J Rheumatol.

[31] Slobodin G, Nakhleh A, Rimar D, Wolfson V, Rosner I, Odeh M (2016). Increased aortic wall thickness for the diagnosis of aortitis: a computed tomography-based study. Int J Rheum Dis.

[32] Vlachou PA, Khalili K, Jang HJ, Fischer S, Hirschfield GM, Kim TK (2011). IgG4-related sclerosing disease: autoimmune pancreatitis and extrapancreatic manifestations. Radiographics.

[33] Rudd JH, Myers KS, Bansilal S, Machac J, Pinto CA, Tong C (2008). Atherosclerosis inflammation imaging with 18F-FDG PET: carotid, iliac, and femoral uptake reproducibility, quantification methods, and recommendations. J Nucl Med.

[34] Besson FL, de Boysson H, Parienti JJ, Bouvard G, Bienvenu B, Agostini D (2014). Towards an optimal semiquantitative approach in giant cell arteritis: an (18)F-FDG PET/CT case-control study. Eur J Nucl Med Mol Imaging.

[35] Martinez-de-Alegria A, Baleato-Gonzalez S, Garcia-Figueiras R, Bermudez-Naveira A, Abdulkader-Nallib I, Diaz-Peromingo JA (2015). IgG4-related disease from head to toe. Radiographics.

[36] Hamano H, Arakura N, Muraki T, Ozaki Y, Kiyosawa K, Kawa S (2006). Prevalence and distribution of extrapancreatic lesions complicating autoimmune pancreatitis. J Gastroenterol.

[37] Fujinaga Y, Kadoya M, Kawa S, Hamano H, Ueda K, Momose M (2010). Characteristic findings in images of extra-pancreatic lesions associated with autoimmune pancreatitis. Eur J Radiol.

[38] Guma M, Firestein GS (2012). IgG4-related diseases. Best Pract Res Clin Rheumatol.

[39] Kaji R, Takedatsu H, Okabe Y, Ishida Y, Sugiyama G, Yonemoto K (2012). Serum immunoglobulin G4 associated with number and distribution of extrapancreatic lesions in type 1 autoimmune pancreatitis patients. J Gastroenterol Hepatol.

[40] Lee TY, Kim MH, Park Do H, Seo DW, Lee SK, Kim JS (2009). Utility of 18F-FDG PET/CT for differentiation of autoimmune pancreatitis with atypical pancreatic imaging findings from pancreatic cancer. AJR Am J Roentgenol.

[41] Ogawa M, Ishino S, Mukai T, Asano D, Teramoto N, Watabe H (2004). (18)F-FDG accumulation in atherosclerotic plaques: immunohistochemical and PET imaging study. J Nucl Med.

[42] Barp A, Fedrigo M, Farina FM, Lepidi S, Causin F, Castellani C (2016). Carotid aneurism with acute dissection: an unusual case of IgG4-related diseases. Cardiovasc Pathol.

[43] Inoue D, Yoshida K, Yoneda N, Ozaki K, Matsubara T, Nagai K (2015). IgG4-related disease: dataset of 235 consecutive patients. Medicine (Baltimore).

[44] Perugino CA, Wallace ZS, Meyersohn N, Oliveira G, Stone JR, Stone JH (2016). Large vessel involvement by IgG4-related disease. Medicine (Baltimore).

[45] Mizushima I, Inoue D, Yamamoto M, Yamada K, Saeki T, Ubara Y, et al. Clinical course after corticosteroid therapy in IgG4-related aortitis/periaortitis and periarteritis: a retrospective multicenter study. Arthritis Res Ther. 2014;16:R156. doi:10.1186/ar4671. 10.1186/ar4671 PMC4220557 25056443

